# Myocardial dysfunction in children with intrauterine growth restriction: an echocardiographic study

**DOI:** 10.5830/CVJA-2016-053

**Published:** 2017

**Authors:** Katazyna Niewiadomska-Jarosik, Justna Zamojska, Agata Zamecznik, Jerzy Stańczyk, Agnieska Wosiak, Piotr Jarosik

**Affiliations:** Department of Pediatric Cardiology and Rheumatology, 2nd Chair of Pediatrics, Medical University of Lodz, Poland; Department of Pediatric Cardiology and Rheumatology, 2nd Chair of Pediatrics, Medical University of Lodz, Poland; Department of Pediatric Cardiology and Rheumatology, 2nd Chair of Pediatrics, Medical University of Lodz, Poland; Department of Pediatric Cardiology and Rheumatology, 2nd Chair of Pediatrics, Medical University of Lodz, Poland; Institute of Information Technology, Technical University of Lodz, Poland; Department of Pediatric Cardiosurgery, Polish Mother’s Memorial Institute, Lodz, Poland

**Keywords:** echocardiography, Intrauterine growth restrictions, Myocardial dysfunction, Children

## Abstract

**Introduction:**

The prevalence of intrauterine growth restriction (IUGR) is about 3–10% of live-born newborns and can be as high as 20% in developing countries. It may result in the occurrence of cardiovascular diseases later in life.

**Methods:**

The aim of this study was echocardiographic evaluation, with the use of conventional and tissue Doppler parameters, of cardiac function in children born with IUGR, and comparison with healthy peers born as normally grown foetuses.

**Results:**

In the IUGR group, E wave and E/A ratio were significantly lower compared to the control group. A wave, isovolumetric relaxation time, deceleration time, myocardial performance index as well as E/E′ septal and E/E′ lateral indices were significantly higher compared to healthy peers.

**Conclusion:**

Children with IUGR presented with subclinical myocardial dysfunction.

## Introduction

Intrauterine growth restriction (IUGR) is a major problem in present-day medicine. It is defined as a birth weight below the 10th percentile for gestational age and involves about 5–10% of neonates.^1^ IUGR is one of the main causes of low birth weight and directly affects perinatal morbidity and mortality rates.

It is well known that such pathology may be associated with the later occurrence of cardiovascular diseases. Barker’s hypothesis, published in 1989,^2^ proved increased incidence of cardiovascular diseases in adults born with IUGR, particularly hypertension and hyperlipidaemia. It is explained by the ‘foetal programming’ theory, which is the formation in the prenatal period of adaptive mechanisms to prevent the long-term hypoxia accompanying IUGR.^3^ Currently, cardiovascular dysfunction forming during the prenatal period is considered one of the main pathophysiological features of this programming.^4^-^6^ In addition, Crispi et al. suggest that this dysfunction may be one of the major mechanisms explaining increased cardiovascular mortality rates in adults who were born with IUGR.^4^

Due to the sparsity of reports, the increase in incidence of myocardial dysfunction in children born with IUGR remains unclear. The aim of our prospective study was echocardiographic evaluation of cardiac function in children born with IUGR, compared to children born at normal gestational age and birth weight.

## Methods

The analysis included 77 children (42 girls, 35 boys), aged from 5–11 years, who were randomly selected from the obstetrics and gynecology out-patient clinic. We included those born at term as small-for-gestational-age (SGA) babies (birth weight below the 10th percentile according to gestational age) with IUGR features, detected prenatally by foetal size measurements on obstetric ultrasonography. All the children were single births. The control group included 30 healthy subjects (16 girls, 14 boys), born with normal birth weight, gender and age matched to the study group. Gestational age was calculated from the mother’s last menstrual period.

All patients were hospitalised at the Pediatric Cardiology and Rheumatology Department of the Medical University of Lodz between 2010 and 2013. All demographic and anthropometric data were recorded during the examination, including information about gestational age, birth weight and nutritional status [height, weight, body mass index (BMI) = weight (kg)/height (m)^2^] (Table 1). All subjects were well at the time of the study, none had a chronic illness or a history of medication taking.

**Table 1:** Patients’ characteristics

**	*Study group (n= 77)*	*Control group (n= 30)*	**
*Characteristics*	*mean ± SD*	*mean ± SD*	*p-value*
Gender (M/F)	35/42	14/16	NS
Age on examination	7 y 8 m ± 1 y 4 m	7 y 7m ± 1 y 10 m	NS
Birth weight (g)	2541.62 ± 218.47	3394.33 ± 522.35	< 0.001
Gestational age (hbd)	39.03 ± 0.90	39.07 ± 0.74	NS
Height (cm)	124.46 ± 10.40	129.30 ± 10.27	< 0.05
Weight	25.20 ± 9.10	27.20 ± 8.10	NS
BMI	15.87 ± 3.01	16.09 ± 2.60	NS

n, number of children; SD, standard deviation; NS, not significant; M, male; F, female; y, years; m, months; hbd, weeks of gestation; BMI, body mass index.

The exclusion criteria were: evidence of chromosomal or infectious aetiology for IUGR, gestational diabetes, hypothyroidism, systemic or acute disease, and the mother smoking cigarettes or using medication. This study was approved by the medical ethics committee of the Health Sciences Faculty of Lodz University (No: RNN/150/09/KB).

All patients underwent a full echocardiographic study, using the Aloka Prosound α 10 device, evaluating anatomy and cardiac function. In standard projections, systolic and diastolic function of the left ventricle was estimated.

Left ventricular diameter, ejection fraction (EF) and shortening fraction (SF) were calculated in the parasternal, long-axis view, in M-mode presentation according to the Teichholz formula.
In the apical, four-chamber view, with the use of pulsed Doppler, evaluation of mitral inflow velocities was done. The sample volume was placed at the mitral valve annulus and the peak of early mitral inflow velocity (E wave in early diastole), the peak of atrial mitral inflow velocity (A wave in atrial systole) and the E/A ratio were assessed.
In the apical, five-chamber view, with the use of pulsed Doppler, left ventricular inflow and outflow were recorded with the sample volume placed between the aortic and mitral valves to evaluate isovolumetric relaxation time (IRT) and deceleration time (DecT). On the basis of these measurements, myocardial performance index, defined as quotient of the sum of the isovolumetric contraction time (ICT) and IRT-to-left ventricular ejection time (ET), were calculated.
In the apical, five-chamber view, with the use of pulsed Doppler, aortic flow was recorded and heart rate calculated.

Each measurement was obtained for three cardiac cycles. For statistical analysis the mean value was used.

Pulsed and colour tissue Doppler imaging were performed in the apical four-chamber view. The sample volume was positioned as parallel as possible with the lateral mitral annular motion. The maximal myocardial velocities during systole (S), and early (E′) and late (A′) diastole were measured at the interventricular septum (septal) and lateral annulus (lateral). The ratio of the early to late diastolic velocities was calculated for IVS (E′/A′ septal) and for the posterior wall (E′/A′ lateral). The ratio of peak transmitral E velocity to early diastolic mitral annular velocity (E/E′) was calculated for both the interventricular septum and posterior wall (E/E′ septal and E/E′ lateral, respectively).

## Statistical analysis

Descriptive statistics were executed by computing the mean and standard deviation (SD) for scale variables, or frequencies for nominal variables. The significance level was computed for the differences between variables in the IUGR and control groups. To evaluate the differences between the two groups, a parametric t-test and a non-parametric Mann–Whitney test were performed. Distributions were checked for normality with the Kolmogorow–Smirnov test. Statistical significance was defined as a p-value < 0.05. Pearson and Spearman correlation coefficients were computed to evaluate the degree of association between variables for either the control or study group.

## Results

Analysis of the medical records confirmed statistically significant differences in birth weight (IUGR group: 2 541.62 ± 218.47 g versus control group: 3 394.33 ± 522.35 g; p < 0.001), while there was no significant difference for gestational age between the groups. Physical examinations did not reveal statistically significant differences between the mean values for weight and body mass index, whereas in the IUGR group, the children were significantly smaller compared to healthy subjects (Table 1).

Analysis of echocardiographic left ventricular diameters did not reveal any significant differences in diastolic wall dimensions (IVSd: interventricular septum in diastole, LVPWd: posterior wall diameter in diastole) or left ventricular lumen diameters between the two groups. Left atrial diameter also did not differ significantly.

The mean values of ejection fraction as well as shortening fraction were within normal limits and similar between the groups. The mean values were as follows: IUGR group: EF 69.32 ± 2.99%, SF 38.33 ± 2.55%; control group: EF 69.20 ± 3.14%, SF 38.30 ± 2.73%.

According to parameters evaluating left ventricular diastolic function, there were statistically significant differences between mean values of E wave, A wave and E/A ratio. The mean values of E wave and E/A ratio were significantly lower in children with intrauterine growth retardation compared to the control group. The mean values of the A wave were significantly higher in that group of patients

The mean values of isovolumetric relaxation time and deceleration time were significantly higher in children with IUGR compared to the healthy peers (102.47 ± 8.72 vs 96.58 ± 6.08 min; 180.81 ± 38.69 vs 160.83 ± 25.63 min, respectively). The mean values of myocardial performance index obtained in the IUGR group were significantly higher than those of the control group. The heart rate was similar in both groups of patients (p = 0.095) (Table 2).

**Table 2:** Echocardiographic parameters

**	*Study group (n= 77)*	*Control group (n= 30)*	**
*Parameters*	*mean ± SD*	*mean ± SD*	*p-value*
LVDd (mm)	37.23 ± 4.27	37.03 ± 3.96	0.81 (NS)
IVSd (mm)	5.40 ± 0.89	5.36 ± 0.85	0.78 (NS)
LVPWd (mm)	5.93 ± 0.91	5.92 ± 0.91	0.95 (NS)
EF (%)	69.32 ± 2.99	69.20 ± 3.14	0.86 (NS)
SF (%)	38.33 ± 2.55	38.3 ± 2.73	0.95 (NS)
E (cm/s)	91.71 ± 14.99	101.07 ± 10.59	0.002
A (cm/s)	68.37 ± 9.91	44.78 ± 9.15	< 0.001
E/A ratio	1.44 ± 0.13	2.01 ± 0.29	< 0.001
IRT (ms)	102.47 ± 8.72	96.58 ± 6.08	0.002
DecT (ms)	180.81 ± 38.69	160.83 ± 25.63	0.011
MPI	0.58 ± 0.08	0.43 ± 0.09	< 0.001
HR/min	85.00 ± 11.41	81.77 ± 8.94	0.095 (NS)

n, number of children; SD, standard deviation; NS, not significant; LVDd, left ventricular diameter in diastole; IVSd, interventricular septum in diastole; LVPWd, posterior wall diameter in diastole; EF, ejection fraction; SF, shortening fraction; E, E wave; A, A wave; IRT, isovolumetric relaxation time; DecT, deceleration time; MPI, myocardial performance index; HR, heart rate.

Estimation of left ventricular function using tissue Doppler imaging did not reveal significant differences between the mean values of systolic myocardial velocity, either at the level of IVS, or at the posterior wall. The mean values of septal and posterior wall E′/A′ ratio were also similar. The mean values of E/E′ for the interventricular septum and E/E′ for the posterior wall were significantly higher in patients with IUGR (Table 3).

**Table 3:** Tissue Doppler echocardiography parameters

**	*Study group (n= 77)*	*Control group (n= 30)*	**
*Parameters*	*mean ± SD*	*mean ± SD*	*p-value*
S septal	7.71 ± 1.20	7.88 ± 1.43	0.58 (NS)
S lateral	9.62 ± 1.37	9.90 ± 1.57	0.40 (NS)
E′ septal	15.29 ± 1.88	14.80 ± 1.83	0.22 (NS)
A′ septal	6.71 ± 1.41	6.32 ± 1.01	0.17 (NS)
E′ lateral	20.38 ± 2.94	20.85 ± 2.24	0.43 (NS)
A′ lateral	7.76 ± 1.86	7.56 ± 0.94	0.56 (NS)
E′/A′ septal	2.41 ± 0.40	2.36 ± 0.43	0.59 (NS)
E′/A′ lateral	2.82 ± 0.42	2.75 ± 0.54	0.51 (NS)
E/E′ septal	6.89 ± 1.11	4.62 ± 1.16	< 0.001
E/E′ lateral	6.10 ± 1.27	4.91 ± 0.88	< 0.001

n, number of children; SD, standard deviation; NS, not significant; S septal, myocardial velocity during systole at interventricular septum; S lateral, myocardial velocity during systole at posterior wall; E′/A′ septal, ratio of early to late diastolic velocities for the interventricular septum; E′/A′ lateral, ratio of early to late diastolic velocities for the posterior wall; E/E′ septal, ratio of peak transmitral E velocity to early diastolic mitral annular velocity for the interventricular septum; E/E′ lateral, ratio of peak transmitral E velocity to early diastolic mitral annular velocity for the posterior wall.

## Discussion

In recent years, more attention has been paid to changes in cardiac function in children with features of intrauterine growth retardation. Many articles describe the disorders appearing as early as in the foetus, revealing subclinical changes in the myocardium detected on echocardiographic examination.^3^,^4^,^7^ Changes in utero due to chronic hypoxia and malnutrition, with increased placental vascular resistance, result in pressure and volume overload of the foetal heart, which in turn induces abnormal cardiac function.^8^,^9^

In our study we evaluated the cardiac function in small-forgestational- age children with features of IUGR with the use of conventional as well as tissue Doppler echocardiographic parameters. We analysed left ventricular diameters (IVSd, LVPWd and LVDd) but did not find significant differences between the groups. This is similar to the findings of Bjarnedard et al. and Crispi et al., who studied, respectively, young adults and children the same age as our study patients.^7^,^10^

On the other hand, Turkish authors demonstrated that left ventricular diastolic dimension in neonates and infants were significantly higher in children with IUGR.^11^ These differences may have been due to the different age groups examined by the researchers.

Other conventional echocardiographic parameters assessed in our study were shortening fraction (SF) and ejection fraction (EF) of the left ventricle. As in other reports, there were no differences between the groups.^7^,^11^,^12^

Altin et al. describes a significantly higher heart rate in children with growth restriction, which was not observed in our study.^11^ Presumably this difference may have been due to the fact that the authors evaluated neonates and infants, not older children, as in our study. These observations may indicate that predominance of the sympathetic nervous system in children with SGA tends to disappear with age. However, Crispi et al. studied children of a similar age as ours and showed similar heart rates to ours in children with IUGR.^7^

E/A ratio is a conventional index evaluating ventricular filling during diastole. In adults, reduced E/A ratios indicate diastolic dysfunction. Cohen et al. measured a mean E/A index of 1.9 in healthy people over 21 years of age and Eto et al. of 1.77 ± 0.53 in children. We had similar findings in the control group, but in children with IUGR, the mean E/A ratio was lower.^13^,^14^

Other articles on children with IUGR describe different values for this index. In most recent reports in foetuses, the E/A ratio was significantly higher compared with the control group. In older children authors describe a normalisation of the E/A ratio, with no differences between a group of healthy children and those born with IUGR features.^7^,^10^,^11^,^15^,^16^

The lower E/A index in children with IUGR seems to be dependent on the increase in A wave and decrease in E wave in those patients. This could be explained by a lower free inflow (E wave) than active left ventricular filling (A wave), which is associated with susceptibility of the left ventricle to abnormalities, and may be one of the symptoms of diastolic dysfunction. Such abnormalities are described in children with connective tissue diseases, due to the higher content of connective tissue in cardiac muscle, which diminishes its susceptibility to stretching.

The results of research on deceleration time in adults, which is an indicator of left ventricular ‘stiffness’, support this theory.^17^ In our study, deceleration time in the IUGR group was only slightly higher but a statistically significant difference was observed between the groups. It may indicate the start of myocardial changes in susceptibility/stiffness of the left ventricle.

With regard to the IRT, results in the literature are not consistent. Sehgal et al., who studied a slightly younger group of children with growth restrictions, described a similar tendency to our results.^18^ By contrast, Crispi et al., in a similar age group to ours, did not report such differences.^7^

In our study, MPI was significantly higher in the IUGR group.^11^,^19^ Similar observations have been reported in foetuses, which may indicate that myocardial function is already impaired in utero.^20^ However, observations from Swedish authors who studied adults who were born with IUGR (22–25 years old) did not confirm this theory.^10^ These differences may have been due to the degree of restriction abnormalities, which was different in the various groups studied.

There are few reports on diastolic cardiac function in children with IUGR. Altin et al. evaluated diastolic function in neonates and infants and revealed that indices that were abnormal in the foetal period (E′, A′, E′/A′, E/E′ septal and E/E′ lateral) tended to decrease with age.^11^ Similar findings were described by Bjarnegard et al., who estimated diastolic function in young adults. They found no differences in these indices between the IUGR and control groups.^10^ On the other hand, a multicentre study by Crispi et al. showed different results.

Other researchers evaluated a similar age group to those in our study and, as in our analysis, some diastolic function parameters were significantly higher in IUGR children. Perhaps the observed anomalies due to intrauterine chronic hypoxia leading to cardiac volume overload and cardiac remodelling resulted in impaired cardiac function.^7^

## Conclusion

From our results, we found that diastolic function may be impaired in IUGR patients, but further studies with larger sample sizes are needed. This group of patient should be monitored long term and evaluated for cardiovascular status, due to the high risk of cardiovascular disease in adulthood.
